# How mothers of a child with type 1 diabetes cope with the burden of care: a qualitative study

**DOI:** 10.1186/s12902-022-01045-z

**Published:** 2022-05-13

**Authors:** Yusef Haghighi Moghadam, Zhaleh Zeinaly, Fatemah Alhani

**Affiliations:** 1 Faculty of Nursing & Midwifery School, Urmia University of Medical Sciences, UMSU Central Site: Orjhans Street, Resalat Blvd, Urmia, 571478334 Iran; 2 Faculty of Medical Sciences, Department of Nursing, Tarbiat Modares University, Tehran, Iran

**Keywords:** Diabetes, Coping, Caregiver burden, Children, Mother

## Abstract

**Introduction:**

Caregiver burden is a complex construct that depends heavily on the context and culture of the community in which care takes place. This study aimed to explore the lived experience of being mothers of a child with type 1 diabetes aged 6 to 18 years.

**Materials and methods:**

We used a qualitative methodology utilizing conventional content analysis. We conducted 24 interviews with 20 mothers who had a child with type 1 diabetes aged 6 to 17 years.

**Results:**

The mean age of mothers and children were 36.3 and 12.3 years, respectively. The mean of years with the disease was 4.3 years. Thirteen children were girls. The essential theme was coping with the burden of care through personalized coping and active acquisition of social support. The main theme consists of four sub-themes including Crisis in the family and burden of care, Losing the family equilibrium, Personalized coping strategies, and Active acquisition of social support. Mothers used personalized strategies and every support they could get to reach their aim.

**Conclusions:**

Families of children with type 1 diabetes need extensive and personalized care plans.

## Introduction

Type 1 diabetes (T1D) is a chronic autoimmune disease characterized by a deficiency of insulin. Insulin deficiency is due to pancreatic β-cell loss and it leads to hyperglycemia [1]. The Middle East and North Africa (MENA) have the highest worldwide prevalence of diabetes at 10.9%. In Iran, it is estimated that the national prevalence of diabetes was 11.37% of the adult population in 2011 which showed a 35% increase from 2005 [2]. With this increase in rate by the year 2030, 9.2 million Iranian individuals will have diabetes [3, 4]. The results of a study estimated that 11.4% of all cases of diabetes in Iran are type 1 [5].

The presentations of diabetes in children with type 1 diabetes are polyuria, polydipsia, weight loss, and diabetic ketoacidosis [6]. This type of diabetes is most common in children and adolescents [7]. The diagnosis of diabetes in children is a very stressful situation for parents. Management of this disease requires a rigorous and complex regimen that includes continuous monitoring and constant attention from caregivers especially mothers [8, 9]. This rigorous care program, along with other negative effects of diabetes on family relationships, can place a heavy caregiver burden on the mother.

Caregiver burden is a complex construct that depends heavily on the context and culture of the community in which care takes place [10]. There are several definitions of this concept. It is defined as the distress that caregivers experience as a result of providing care [11]. It is also defined as the physical, psychological or emotional, social, and financial problems that can be experienced by the members of the family caring for an impaired person [12]. For the family to be able to return to normal functioning, it is necessary for the parents, especially the mother, to adapt to the burden of care [11]. In Middle Eastern countries such as Iran, mothers are usually responsible for caring for their children. Working women are more likely to care for children than men [13].

Some studies have examined the caregiver burden of parents or mothers with children with diabetes. For example, a study on blog posts and their associated comments were analyzed using thematic analysis and five major themes emerged. They were respectively the impact of the child’s diagnosis, the burden of intense self-management experienced in caring for a child with T1D, caregivers’ use of technology to ease their fear of hypoglycemia, and impacts that device alarms associated with this technology have on caregiver burden, caregivers’ perceptions of frequently missed or delayed diagnosis of T1D and the frustration this causes, and the resilience that caregivers develop despite the burdens they experience [14]. Another study in China showed that family members were the main care providers for pediatric T1DM patients. They need to handle a diverse variety of problems related to caring [15]. Based on their results problems of caring cause a lot of caregiver burden. Participating mothers in a study by Rossiter, Cooper [16] in the United Arab Emirates described initial reactions of shock and disbelief to the diagnosis of diabetes in the children. While the initial reaction was followed by near ordinary and normal family functioning, they found that there is a lot of burden of care in these children. The family, culture, and faith as critical supports in the whirlwind daily challenge of balancing the multiple demands and competing needs of the newly diagnosed child and the broader family. A study in India showed that parents had depressive symptoms regarding self-reported T1D stigma which was related to poor glycemic control [17]. In summary, caregiver burden is a big concern of families with children with diabetes. It can cause mental health problems and also is related to several factors like social support.

According to previous literature, caring for children with diabetes is a very difficult task. Mothers are the primary caregivers in most countries, especially in the middle eastern ones and cultures [10, 18]. Most mothers and families accept the disease eventually but in different paths. Our insight about the burden of care and how mothers deal with it can help us provide better care in different stages of their coping process. The study aimed to explore the experience of mothers who had a child with Type 1 diabetes from receiving the diagnosis and caring for their child.

## Methods

This study was qualitative research that was performed using conventional content analysis. The use of content analysis as a research method has been widened in health studies in recent years. Conventional content analysis aims to describe a phenomenon [19–21]. The phenomenon in this study was the lived experience of mothers of a young child with T1D.

### Participants

The study participants were 20 mothers of a child with T1D. The inclusion criteria consisted of mothers who had a child with T1D, were Iranian, married, living with their spouse, had no physical or mental problems, at least one year had passed since their child was diagnosed with T1D, and willing to participate in the study. The recruitment was conducted at two centers including the Pediatric hospital of Urmia, Iran, and Children's Medical Center of Tehran, Iran from June 2019 to June 2020. The participants were recruited using a flyer with study aim and details asking mothers of a child with T1D to speak with the researcher (First Author) on the subject of their experience of diabetes diagnosis and care. Purposeful sampling was applied and at the end, a total of 24 mothers were invited. Of them, 20 mothers gave their consent to participate. The study participants' demographic characteristics are presented in Table 1. Four mothers did not have enough time to participate in interviews.

**Table 1:** Demographic characteristics of study participants

ID	Age	Siblings	Education	Job	Age of Child (Year)	Birth Rank	Gender	Disease Duration (year)
1	31	2	Elementary School	Housewife	13	1	Female	7
2	44	3	Elementary School	Housewife	12	3	Female	3
3	35	2	High School	Housewife	14	2	Male	1
4	35	3	Elementary School	Housewife	12	3	Female	6
5	40	4	Illiterate	Housewife	15	2	Female	8
6	38	1	Diploma	Housewife	14	1	Female	13
7	30	2	Diploma	Housewife	13	1	Female	1.5
8	38	2	College Degree	Retired Employee	11	2	Female	1.5
9	31	1	Diploma	Housewife	10	2	Male	2
10	40	3	Illiterate	Housewife	13	2	Female	1.5
11	48	4	High School	Housewife	13	4	Male	1.5
12	38	3	Elementary School	Housewife	7	3	Female	5
13	34	2	Elementary School	Housewife	12	1	Female	6
14	32	2	Diploma	Housewife	14	1	Male	2
15	20	3	Elementary School	Housewife	13	3	Male	10
16	33	2	High School	Housewife	6	2	Male	5
17	32	2	Illiterate	Housewife	13	1	Female	2
18	42	2	Diploma	Housewife	12	1	Female	3
19	47	3	Elementary School	Housewife	11	3	Male	1
20	38	1	College Degree	Employee	16	1	Female	6

### Data collection

Data were collected through in-depth semi-structured interviews conducted by the first author who is a Ph.D. candidate in nursing. The first author attended several qualitative study workshops and the interviews were guided by one supervisor and one advisor. Twenty-four interviews with twenty participants were conducted. First, the aim and procedures of the study were explained to all participants. Written informed consent was obtained and the interviews were recorded by an mp3 recorder. The interviews began with open-ended questions like “Tell me about your caring experience of your child?”, “How did you find out about your child's diabetes?”, “How has your child's diabetes affected your life?”. Follow-up questions continued on the mothers’ responses. With 21 interviews the data got redundant and all aspects of the main theme and subthemes emerged. Three more interviews were conducted to ensure that the data saturation was achieved. The duration of each interview was 20–84 min. To create maximum variation, participants were selected from different social, economic, and educational levels.

### Data analysis

MAXQDA software (version 10, VERBI Software, Berlin, Germany) was used for the transcription and classification of codes. Data were analyzed using conventional content analysis. The first author transcribed all interviews verbatim. Then she read them repeatedly to obtain a general idea. In the first step of coding, the meaning units were determined and compressed into initial codes. The meaning units were the whole interview, paragraphs, and sentences. The initial coding was conducted by the first author (A nursing Ph.D. candidate) and supervised by one supervisor and one advisor who are Ph.D. in nursing and expert in the field of qualitative research. Codes were placed in subcategories based on their similarity and differences and each of these subcategories formed categories. In the end, the main categories that provide a pattern formed the main theme which consisted of four sub-themes (Graneheim & Lundman, 2004). The data analysis started with the first interview, and future interviews were guided by the analysis and this process continued until the last interview (Elo & Kyngas, 2008; Graneheim & Lundman, 2004). The analysis moved back and forth between the main theme and sub-themes until consensus was reached by all authors.

### Rigor

Guba & Lincoln criteria, including credibility, transferability, conformability, and dependability were used to assure the rigor of qualitative data [19, 22]. Member checking was carried out to ensure the data's credibility. All codes were checked with participants after interviews by phone or email and two extra interviews were conducted with two participants. During the interview, the interviewees’ words were feedback to them for confirmation. Prolonged engagement, sufficient time allocation, and good communication were used as well. We used detailed and thick data descriptions to ensure data transferability therefore other researchers can have a full understanding of research steps. Three experts in the field of qualitative research and diabetes care were asked to review the reports and comments on the codes and findings to achieve conformability. The interviews were conducted using the Persian language. All quotes and questions in this article were translated to English by two members of the research team. After translation, all of them were reviewed with a bilingual expert who is an expert in the English language.

### Ethics considerations

The study was approved by the Ethics Committee and performed in accordance with the Helsinki Declaration. The study was approved by both hospitals' relevant authorities. The mothers were informed that they could withdraw from the study at any time.

## Results

Study participants were 20 mothers who had a child with type 1 diabetes. The main emerged theme was coping with the burden of care through the personalized coping and active acquisition of social support. The main theme consisted of four sub-themes including Crisis in the family and burden of care, Losing the family equilibrium, Personalized coping strategies, and Active acquisition of social support. The main theme, sub-themes, and categories are presented in Table 2.

**Table 2:** The main theme, sub-themes, and categories

Theme	Sub-themes	Categories
Coping with the burden of care through personalized strategies and active acquisition of social support	Crisis in the family and burden of care	Sudden onset High level of stress
		Complex care Rigorous care
	Losing the family equilibrium	Economic imbalance
		Imbalance in the family relationships
	Personalized coping strategies	Self-empowerment
		Changing climate family
		Relationship Making
		Screening the people for relationship
	Active acquisition of social support	Family support
		Organizational support
		Spiritual Support

### Coping with the burden of care through personalized strategies and active acquisition of social support

Being a mother with a child with T1D was a very challenging task. Mothers usually were the first-line caregiver. They considered themselves as the main person, responsible for their child's health. They also had other responsibilities such as caring for other children, job or household tasks, and familial and social relationships that could compromise with the full-time care of a diabetic child. This could also increase the burden of care. They wanted to maintain their family equilibrium. They tried to cope with the diagnosis and its consequences. Also, they sought actively family and social support.

### Crisis in the family and burden of care

Usually, the family found about the T1D diagnosis suddenly and it was very shocking. Hypoglycemia or other emergencies that they had been experienced for the first time were very frightening events. Mothers expressed different emotions when they talked about the time that the child was first diagnosed with diabetes such as fear, grief, shock, and anger. The chronic nature of diabetes and its lifetime complications made it very hard to accept especially for mothers. The family also experienced a time of tension and crisis. The illness of a family member, especially a child, was one of the most difficult situations that family members may face. Family members may have blamed themselves for the illness. Other members of the family depended on the mother’s ability to manage the situation. The child did not have yet reached a level of understanding to know the disease. He/she knew there is a problem but did not know what or why. Most of the responsibility for the emotional support of the child was on the mother. One of the mothers said that: “The first time I noticed a bad smell in his mouth. I was very scared. I realized there is a serious problem.” Another mother explained her feelings as: “I was very sad. The fact that my child will not have a normal life bothered us a lot. His father and sister were also very upset. I had to comfort them.”

The problems of children with treatment were explained by a mother: “She did not understand why she should inject insulin regularly or why she should not eat certain foods. She nodded constantly. I was always with her and I had to support her emotionally.”

These were the short-term consequences of diagnosing diabetes in a child for the family and mothers. Mothers at this stage mostly reported feelings of denial and anger. Then they came to terms with the fact that their child has diabetes. One mother explained her feeling as: “I could not believe that my child had diabetes. I took him to different doctors. I finally accepted that my child had diabetes. I had to deal with it.”

### Losing the family equilibrium

The diagnosis of diabetes in a child disrupted the dynamics of the family. There were long-term consequences for the child and the family. Caring for a diabetic child required constant attention and adherence to a complex medical and dietary regime. At the same time, the mother’s interpersonal relationships within and outside the family were affected. Caring for a child with diabetes had the greatest impact on a mother's occupation. It was very difficult to leave a child to kindergarten or with grandparents. So, in many cases, the mother had to quit her job. This also reduced the family income. Paying more attention to one child may has been hurt the feelings of other children. All of the above-mentioned could harm the balance inside the family. The mothers have been tried to restore this balance in any way possible. One mother said that: “I could not go to work. There were also medicines needed by the child. We also had specialist visits regularly. Our lives were completely out of balance financially.” Regarding the dissatisfaction of other children one mother mentioned that: “I spend a lot of time with Ahmed. This has made his siblings dissatisfied.” Family relationships can damage by the burden as one mother explained: “Going to family gatherings has become very difficult. Because we have to watch his diet. My mom and dad expect me to visit them regularly, but I can't. This has made them irritated.”

### Personalized coping strategies

After going through the initial critical stage, the mothers tried to bring balance back to their lives. Each mother used her strategies. The first strategy was self-empowerment. Mothers sought to increase their knowledge of diabetes and care for diabetic patients. Successful implementation of the caregiver role required significant competencies and a positive attitude towards disease control. Mothers started to find new information “I started reading online about diabetes care, I obligated myself to take the responsibility of the care of my child myself”.

The second strategy was the change in the emotional and psychological climate of the family. Creating a happy climate within the family and reducing stress increased blood sugar control and improved interpersonal relationships. As one mother said: “We try to make the atmosphere of the house happy. When we are happy, Ali is happy too and he cooperates better”.

The third strategy was to preserve existing relationships and create new ones. Also, establishing new relationships was especially important for mothers, particularly with healthcare providers. Good relationship with healthcare team was a good support resource: “I established a good relationship with the health center physician. Every time I had a problem, she helped me”.

Reducing the negative interference of relatives was the fourth strategy of mothers. Negative comments can weaken the attitude of the family and the diabetic child. Communicating with hand-picked people who worked to improve the child's mood was another strategy for mothers. As one mother said: “We cut ties with our relatives who gave negative comments. Some people’s words do not help. We interact more with those who have a positive effect on Muhammad's recovery.”

### Active acquisition of social support

The second factor that helped mothers cope with their child's illness was strong social support. Three levels of support were identified, including family, social, and spiritual. Mothers who actively pursued these sources of support were better able to cope with their child’s diabetes and decrease the burden of care.

The family was the first source of support for mothers. Intra-family support, including support from the husband and other children, was the most effective source of support. Mothers actively involved their husbands and other children in the process of caring for a diabetic child. One mother mentioned her husband support: “My husband participates in all the care; he also takes care of the other children. He always supports me emotionally.”

Relatives like grandmothers and grandfathers were another source, but they could not help efficiently because of the complexity of care. Support from relatives was mentioned by mothers: “My mother would love to help, but caring for a diabetic child is very complicated.”

The second source of support was supportive and social groups. These supporters included peers, healthcare providers, and social organizations (insurance organizations, the Diabetes Association, the school), who play an important role in maternal care. As one mother said “The Diabetes Association and the hospital have regular training sessions. I received a lot of information from them. They also have consulting services.”

Given the important role that religion and spirituality play in the lives of Iranians, spiritual support was a strong source of support. Mothers used their spiritual resources in this situation: “I always hoped in God, especially in those critical times. Prayer always gives me hope.”

## Discussion

The current study aimed to explore the experience of mothers with a diabetic child of the coping process with the diagnosis and caregiver burden. Our results showed that mothers of children with T1D can cope with the disease with their personalized strategies and actively get support from proper resources. The process showed a path from crisis to coping. While all mothers can reach a level of coping with their child's diabetes, the strategies that they use, and the social support they can get, help them to decrease the burden of care.

The first encounter with diabetes was found very stressful for mothers. They experienced feelings of anger, denial, and eventual acceptance of their child's illness. Exposure to the disease of a child has also been reported in previous studies as a stressful experience. Bowes, Lowes [23] reported that parents of children with diabetes had feelings associated with grief, such as anger and guilt. They reported that while parents had adapted to the needs of diabetes management, they experienced a resurgence of grief at critical times during their child’s growth. Our results also showed the same crisis situations like when the mother wants to keep her job, they may face with new challenges.

Diabetes, like other chronic diseases, opens a dark horizon for the family. Lifelong illness, the need for insulin injections, and life-threatening and debilitating complications can greatly increase maternal anxiety. The above mentioned can explain the crisis and the sense of helplessness for mothers. The need for constant care and fear of severe complications can also explain the high care burden. T1D is among the diseases with the highest caregiver burden [24]. Numerous studies have shown that the burden of caring for mothers with a child with diabetes drastically reduces their quality of life [25–28]. Even the sleep of the parents of a child with diabetes is severely affected [29–32]. Our results also showed that the nature of diabetes creates a crisis for the family and the burden of caring intensifies it. The results of a study by Commissariat, Harrington [33] showed two types of burden including the emotional burden of diabetes on parents and their children and the burden of finding, training, and trusting effective secondary caregivers. Parents believed that they play the role of child pancreas. Our results also showed the same pattern of facing with the disease and trying to find suitable resources.

Our results showed a crisis in the first steps of diagnosis where the family are in shock. The results of a study in Iran concluded that the reactions at the time of diagnosis were mainly due to lack of information, lack of attention to their needs at the time of diagnosis, and the sudden diagnosis and lack of enough time for mothers to accept the disease [34]. According to our results, the whole family needs support to be able to bring balance back to life.

Our results showed that T1D in a child can push the family out of equilibrium. The family income and relationships within and outside the family will change. While adults with chronic diseases can cope with the new lifestyle, children need their caregivers to do the changes. As the results of a study conducted by Moreira, Frontini [35] showed that caring for a child with Type 1 diabetes can change interfamily relationships, our results showed that the child siblings may experience a feeling of abundance. We think the relationships within the family should be taken under consideration, the finding that was mentioned in another study [36].

Our results showed that because of quitting the job by mother due to the caregiving to the diabetic child can affect the family income and increase its expenses, the results of several studies showed that the family income was related to poor adherence to treatment [37, 38]. Based on our results losing the equilibrium of the family was a threat to the children and parents' health.

Our results showed that mothers tried to overcome the burden of care and regain the balance of their family through personalized strategies and finding new sources of support. This result was a new approach to see the efforts of mothers to cope with their children's disease. Providing care to a child with a chronic disease needs vast sources of support but usually, mothers are alone on it. Families and especially mothers who are successful in the process of overcoming the burden of care use their limited resources more smartly and effectively. This result was in line with another study that was conducted to evaluate the benefits of a Family Strengths Oriented Therapeutic Conversation intervention to mothers of children and adolescents in Iceland with newly diagnosed chronic illnesses/disorders. Their results showed that they reported significantly higher family support, greater conviction about their illness beliefs, increased quality of life, and greater satisfaction with health care services [39]. Our results showed that mothers can benefit from the support of healthcare providers. The results of a study by Khandan, Abazari [40] showed that the mothers of children with diabetes face care management challenges, and they experience a hard life. They concluded that transferring the roles of professional caregivers to mothers can cause many challenges. Our results showed that mothers were using the help of healthcare professionals to reach independence in care.

We found a path that Iranian mothers used to cope with the burden of care of a child with T1D. Our results were unique and relatable to eastern cultures that mothers are mostly the first caregiver and governmental and social supports are usually weak. However, our review showed that in other cultures the burden of care is also high and can change the balance of the family. We also interviewed only mothers with one diabetic child. New studies with mothers who have more children with diabetes can be helpful to fully understand the process of coping.

## Conclusion

Our results showed a path from crisis to cope in mothers who had a child with diabetes. Based on our results mothers in the absence of effective health care and support institutions in Western countries, family members, especially mothers, have a greater role to play in caring for children in developing countries. Contrary to the results of some previous research, our results showed that mothers tried to reduce the burden of care and restore balance to the family by using personalized strategies. Families who have children with type 1 diabetes need extensive and personalized care plans.

## Data Availability

The datasets generated and/or analysed during the current study are not publicly available due the nature of the study. The study is conducetd with a qualitative method. Data are available from the corresponding author on reasonable request.

## Abbreviations

T1D: Type 1 diabetes (T1D)
MENA: The Middle East and North Africa
